# FusionTCN-Attention: A Causality-Preserving Temporal Model for Unilateral IMU-Based Gait Prediction and Cooperative Exoskeleton Control

**DOI:** 10.3390/biomimetics11010026

**Published:** 2026-01-02

**Authors:** Sichuang Yang, Kang Yu, Lei Zhang, Minling Pan, Haihong Pan, Lin Chen, Xuxia Guo

**Affiliations:** 1 Department of Mechatronics Engineering, College of Mechanical Engineering, Guangxi University, Nanning 530004, China; 2211401004@st.gxu.edu.cn (S.Y.); 2511401005@st.gxu.edu.cn (K.Y.); zhanglei@ylu.edu.cn (L.Z.); pan_minling@bbgu.edu.cn (M.P.); hustphh@163.com (H.P.); 2 College of Physical Education, Guangxi University, Nanning 530004, China

**Keywords:** gait prediction, temporal convolutional network, attention mechanism, IMU, causal modeling, exoskeleton control, phase consistency

## Abstract

Human gait exhibits stable contralateral coupling, making healthy-side motion a viable predictor for affected-limb kinematics. Leveraging this property, this study develops FusionTCN–Attention, a causality-preserving temporal model designed to forecast contralateral hip and knee trajectories from unilateral IMU measurements. The model integrates dilated temporal convolutions with a lightweight attention mechanism to enhance feature representation while maintaining strict real-time causality. Evaluated on twenty-one subjects, the method achieves hip and knee RMSEs of 5.71° and 7.43°, correlation coefficients over 0.9, and a deterministic phase lag of 14.56 ms, consistently outperforming conventional sequence models including Seq2Seq and causal Transformers. These results demonstrate that unilateral IMU sensing supports low-latency, stable prediction, thereby establishing a control-oriented methodological basis for unilateral prediction as a necessary engineering prerequisite for future hemiparetic exoskeleton applications.

## 1. Introduction

Stroke and hemiparesis remain among the most prevalent neurological disorders leading to chronic gait impairment and long-term functional asymmetry. According to the World Stroke Organization, more than 12 million individuals suffer a stroke annually, and over half experience persistent gait disturbances that limit daily activities and reduce quality of life [1,2,3,4]. Clinical guidelines emphasize the clinical importance of gait recovery in post-stroke rehabilitation [2], and classic biomechanical analyses describe the characteristic asymmetry and compensatory patterns of hemiparetic gait [3]. Because coordinated motion of both lower limbs is essential for stable walking [4], restoring gait symmetry has long been a key objective in post-stroke rehabilitation. [2], and classic biomechanical analyses describe the characteristic asymmetry and compensatory patterns of hemiparetic gait [3].

Wearable robotic exoskeletons have become an important tool in this field, providing intensive, repeatable, and task-oriented training without heavy dependence on therapist supervision [5,6,7,8,9]. For instance, Esquenazi et al. [10] demonstrated the clinical safety and efficacy of the ReWalk powered exoskeleton, reporting that the system successfully restored ambulatory function in individuals with severe motor impairments. Dollar and Herr [5] reviewed early lower-extremity assistive devices and summarized core challenges in actuation, control, and human−robot interaction, while follow-up reviews by Louie and Eng [6], Young and Ferris [7], and Warutkar et al. [8] examined the clinical translation of powered exoskeletons and emphasized constraints related to usability and patient selection. Over time, the field has shifted from replaying unimpaired trajectories [5] to more adaptive strategies that align robotic assistance with the user’s ongoing movement intent, often through phase-variable or oscillator-based control formulations [11,12,13]. Achieving this alignment depends fundamentally on the system’s ability to estimate or predict gait timing and joint evolution in real time.

However, for neurologically impaired individuals, sensors placed directly on the paretic limb often provide degraded signals due to spasticity, restricted range of motion, orthotic interventions, or inconsistent foot strike patterns [4,8]. These practical constraints motivate unilateral sensing strategies that rely exclusively on the healthy limb. This concept draws inspiration from the natural contralateral coupling in human locomotion [14], suggesting that the healthy limb carries predictive structures that can be leveraged to forecast the trajectory of the affected side. Recently, Huo et al. [15] conducted a randomized controlled trial validating this clinical direction, showing that unilateral lower-limb exoskeleton assistance significantly improved balance and gait recovery in subacute stroke patients.

Despite these promising clinical findings, translating this strategy into robust control faces quantified hurdles. Regarding physiological asymmetry, standard predictive models suffer performance degradation when gait deviations increase. For instance, De Miguel-Fernández et al. [16] demonstrated that the correlation between IMU-derived kinematic metrics and exoskeleton control parameters can drop from strong levels (r>0.9) in mild impairment to poor reliability (near 0.0) in severe cases, highlighting the inability of simple regression baselines to capture pathological non-linearities. Furthermore, regarding computational feasibility, state-of-the-art sequence models like the Transformer are computationally intensive for embedded exoskeleton controllers. The base Transformer architecture requires over 65 million parameters [17], introducing inference latencies incompatible with the strict latency constraints required for real-time assistance. Consequently, existing approaches often struggle to balance the modeling of long-term dependencies with the dual requirements of causality preservation and high parameter efficiency.

In this study, we focus on the methodological feasibility of unilateral IMU-based contralateral gait prediction under controlled conditions. The proposed FusionTCN−Attention model is evaluated using gait data collected from healthy adult subjects. This choice serves as a necessary engineering prerequisite to rigorously examine causality preservation and prediction stability, leveraging the inherent gait symmetry of healthy individuals [18] to establish a performance benchmark before clinical deployment. While the ultimate application lies in post-stroke rehabilitation, clinical validation on hemiparetic populations involves distinct pathological challenges and is beyond the scope of the present work.

The main contributions of this study are summarized as follows:

- Biomimetic Architecture:This study introduces FusionTCN−Attention, a causality-preserving model that integrates a dilated TCN backbone with a lightweight temporal-attention module. This design mimics the neural coupling of short-term reflex and long-term rhythm to predict continuous contralateral trajectories using only unilateral IMU inputs.
- Feasibility Validation:The algorithmic feasibility of using six kinematic channels (angle, velocity, acceleration) from the healthy limb to forecast contralateral motion is validated, establishing a necessary engineering benchmark for future hemiparetic applications.
- Comprehensive Evaluation: Experiments demonstrate that the proposed model outperforms conventional sequence models, achieving hip/knee RMSE of 5.71°/7.43° and an average phase lag of 14.56 ms. Hardware-in-the-loop tests further confirm its timing stability for real-time exoskeleton control.

## 2. Related Work

### 2.1. Gait Event Detection and Continuous Prediction

Most existing exoskeleton controllers implement finite-state or event-triggered structures in which the gait cycle is divided into discrete phases such as heel strike, stance, swing, and toe-off [4,9]. These approaches are attractive for their robustness and conceptual simplicity: transitions are triggered using foot-switch signals, force thresholds, or characteristic angular patterns, often detected from kinematic or kinetic features [19,20,21]. Zeni et al. [19] compared simple kinematic rules for detecting heel-strike and toe-off and showed that the chosen rule can shift event timing substantially, while Ghoussayni et al. [20] assessed automated force-based detection and highlighted its sensitivity to noise and threshold selection. Prasanth et al. [21] surveyed wearable-sensor algorithms and concluded that IMU-based detection is feasible across diverse speeds in healthy gait. Nevertheless, phase-variable controllers often degrade when cadence or terrain changes continuously [11,12]. Baek et al. [11] reported reduced performance when gait deviated from nominal patterns, and Best et al. [12] showed that reliable phase estimation is critical for prosthetic knee−ankle control on varying inclines.

Recent deep-learning approaches have improved event detection—Ashraf et al. applied a temporal convolutional network (TCN) to IMU data [22], and Asogwa et al. predicted minimum-clearance events using neural networks [23]—but these studies also note performance gaps under irregular gait. Such limitations have motivated continuous gait modeling approaches that infer joint trajectories or continuous phase directly from sensor streams [24,25,26]. By replacing discrete transitions with continuous predictions, these approaches support smoother reference generation and better responsiveness to natural variations in gait [4,21].

### 2.2. Human–Robot Interaction and Sensing Modalities

In the broader context of human–robot interaction, mapping user intention to robotic control is critical. Su et al. [27] developed a bidirectional teleoperation control system, demonstrating that synchronization between a master and slave system can be achieved through precise kinematic constraints. This offers a theoretical parallel to our approach, where the healthy limb acts as the “master” driving the “slave” exoskeleton. Similarly, Zhou et al. [28] highlighted the importance of multi-modal sensor fusion in decoding human intention with high granularity.

However, deploying such multi-modal systems in daily rehabilitation faces significant practical trade-offs. Vision-based gait recognition systems are often constrained by environmental factors, such as sensitivity to lighting conditions, viewing angles, and potential occlusions, which limit their robustness in unconstrained settings [29]. Conversely, while surface electromyography (sEMG) offers direct neural interfacing, it introduces substantial calibration burdens and signal instability due to electrode shifts, skin impedance variations (e.g., sweating), and muscle fatigue during long-term use [30]. In contrast, IMU-based sensing provides a self-contained, lighting-independent solution with minimal setup requirements [25]. Therefore, we prioritize a unilateral IMU configuration to maximize system portability and deployment simplicity, ensuring the exoskeleton remains suitable for compact, personal use outside strict laboratory settings.

Given these advantages, Inertial Measurement Units (IMUs) have become a preferred low-cost, portable solution [25,31]. Early IMU studies focused primarily on event detection [19,20,21]. Later work extended IMU usage to reconstruct joint kinematics and spatiotemporal parameters [24,25,26]. Favre et al. [26] estimated three-dimensional knee angles in ambulatory settings and showed reasonable agreement with laboratory motion capture. Seel et al. [25] demonstrated robust lower-limb joint estimation without external references, and Li et al. [24] used two shank-mounted IMUs to reconstruct spatiotemporal parameters from a minimal sensor set. Hur et al. [32] trained deep neural networks on open-source IMU datasets and reported that lower-limb joint angles can be recovered across diverse walking conditions. Unilateral sensing strategies, which infer affected-side motion from the healthy side, remain less explored but have shown initial promise in metabolic cost reduction [33] and feasibility monitoring [16].

### 2.3. Temporal Modeling Architectures

To model the predictive structure of gait, recent work has employed deep temporal architectures. Recurrent Neural Networks (RNNs) and their gated variants such as LSTMs and Seq2Seq models have been widely applied to human motion forecasting [34]. For example, Kim and Hargrove [35] proposed an LSTM-based model to predict continuous gait phase for prosthetic legs, achieving high accuracy on benchmark datasets. However, RNNs often suffer from accumulated phase drift over multi-step predictions. Bai et al. [36] showed that temporal convolutional networks (TCNs) can achieve comparable or improved accuracy with more stable training behavior across a range of sequence modeling tasks.

Deep models have also been applied directly to IMU-based gait estimation [22,23,32]. Vaswani et al. [17] introduced the Transformer architecture based on self-attention, which excels at capturing long-range dependencies. However, its standard implementation is ill-suited for wearable robotics due to the quadratic computational complexity of global attention and the lack of strict causal constraints, which complicate real-time inference on resource-limited hardware. As a result, TCNs—which combine dilated causal convolutions with fixed latency—have become increasingly prominent [36,37]. Lea et al. [37] demonstrated the capability of TCNs for structured human activity segmentation, and Ashraf et al. [22] applied a TCN to gait-event detection with robustness to sensor noise. Still, most TCN-based studies focus on classification tasks or assume bilateral sensing, leaving a gap in unilateral trajectory-level gait prediction suitable for exoskeleton control.

## 3. Materials and Methods

### 3.1. Problem Definition

This study targets cooperative exoskeleton assistance by predicting the motion of the affected limb from the kinematic patterns of the healthy limb. Human walking exhibits bilateral coupling with phase-locked dynamics; consequently, the task is cast as a temporal sequence modeling problem in which contralateral dynamics are inferred from the observed motion history of the healthy side under causality constraints and a bounded, deterministic latency budget.

Let the healthy-side kinematic stream be sampled at a constant rate; in practice, raw signals were acquired at 200 Hz and uniformly resampled to $f_{s}=100$ Hz for model training and deployment. Around any time index *t*, a history window of length *N* is defined as

(1) $$X_{t-N+1:t}=\left[x_{t-N+1},\;x_{t-N+2},\;\ldots ,\;x_{t}\right]\in R^{N\times 6},$$

where each frame collects six kinematic features,

(2) $$x_{\tau }={\left[\theta_{\;H}^{\mathrm{hip}}\left(\tau \right),\;{\dot{\theta }}_{\;H}^{\mathrm{hip}}\left(\tau \right),\;{\ddot{\theta }}_{\;H}^{\mathrm{hip}}\left(\tau \right),\;\theta_{\;H}^{\mathrm{knee}}\left(\tau \right),\;{\dot{\theta }}_{\;H}^{\mathrm{knee}}\left(\tau \right),\;{\ddot{\theta }}_{\;H}^{\mathrm{knee}}\left(\tau \right)\right]}^{\;⊤}.$$

where θ, $\dot{\theta }$, and $\ddot{\theta }$ denote joint angle, angular velocity, and angular acceleration, and the subscript *H* indicates the healthy limb. The prediction target is the short-horizon trajectory of the contralateral (affected) hip and knee,

(3) $$Y_{t+1:t+M}=\left[y_{t+1},\;y_{t+2},\;\ldots ,\;y_{t+M}\right]\in R^{M\times 2},\;y_{\tau }={\left[\theta_{\;A}^{\mathrm{hip}}\left(\tau \right),\;\theta_{\;A}^{\mathrm{knee}}\left(\tau \right)\right]}^{\;⊤},$$

with subscript *A* indicating the affected limb. The task is a causal sequence-to-sequence mapping,

(4) $$F:\;\;X_{t-N+1:t}\;⟼\;{\hat{Y}}_{t+1:t+M}.$$

#### 3.1.1. Causality and Deterministic Latency

For cooperative deployment with wearable exoskeletons, the prediction mapping F is required to be strictly causal, such that future motion estimates depend only on information available up to the decision time and do not access any future samples.

(5) $$\begin{array}{cc} {\hat{Y}}_{t+1:t+M} & =F\;\left(X_{t-N+1:t}\right),\\ {\hat{y}}_{\tau } & ={\left[{\hat{Y}}_{t+1:t+M}\right]}_{\tau },\;\forall \;\tau \in \left\{t+1,\ldots ,t+M\right\}, \end{array}$$

This strict causality constraint enables seamless operation on streaming IMU data and supports a fixed, measurable inference delay compatible with controller synchronization.

#### 3.1.2. Control-Oriented Objectives

To maintain biomechanical consistency and closed-loop stability, the predicted trajectories are encouraged to preserve (i) global phase consistency over the gait cycle and (ii) accurate alignment near heel-strike (HS) and toe-off (TO). Let ϕ(·) denote a phase-mapping operator and $\nabla_{t}$ the first-order temporal difference. A practical objective is

(6) $$\begin{array}{cc} \min_{F} & \underset{\mathrm{trajectory}\;\mathrm{accuracy}}{\underset{︸}{∥{\hat{Y}}_{t+1:t+M}-Y_{t+1:t+M}{∥}_{2}^{2}}}\;+\;\lambda \;\underset{\mathrm{temporal}\;\mathrm{smoothness}}{\underset{︸}{∥\nabla_{t}{\hat{Y}}_{t+1:t+M}-\nabla_{t}Y_{t+1:t+M}{∥}_{2}^{2}}}\\ \; & \;+\;\beta \;\underset{\mathrm{phase}\;\mathrm{consistency}}{\underset{︸}{∥\phi \left({\hat{Y}}_{t+1:t+M}\right)-\phi \left(Y_{t+1:t+M}\right){∥}_{2}^{2}}}, \end{array}$$

where λ and β weight smoothness and phase preservation. This formulation serves as a conceptual objective to guide model design, motivating the causal architecture and auxiliary loss terms described in Section 3.3.

### 3.2. Data Acquisition and Preprocessing

#### 3.2.1. Acquisition Protocol

**Figure 1:**
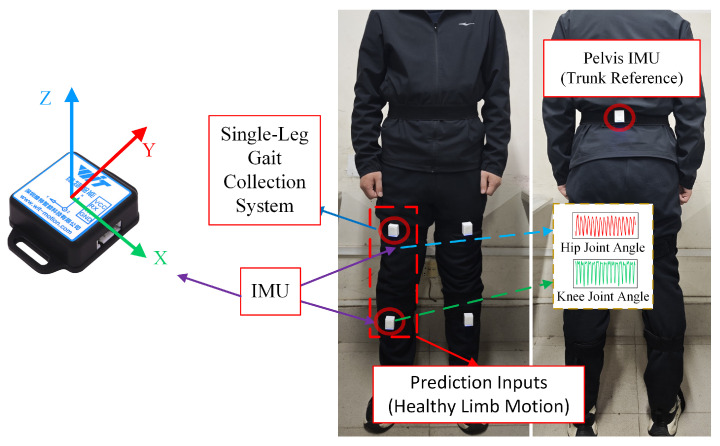
IMU sensor configuration and coordinate definitions on the healthy limb.

**Figure 2:**
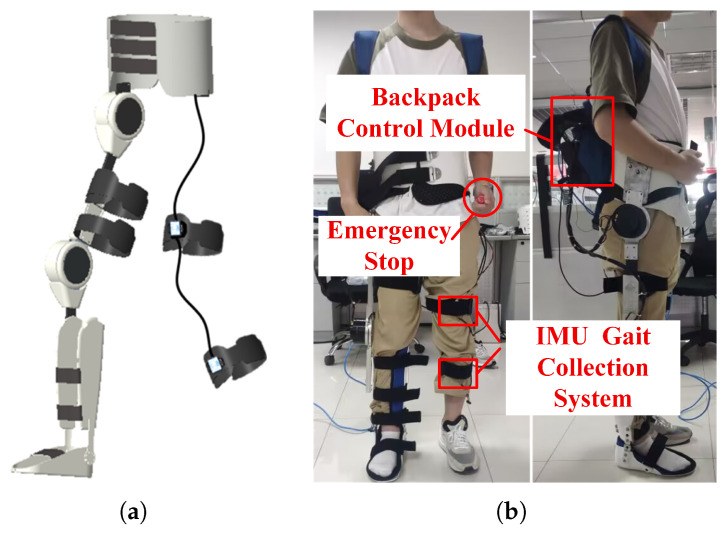
Unilateral lower-limb exoskeleton platform: (**a**) system architecture and (**b**) prototype implementation.

Unilateral lower-limb kinematics were collected using three wearable IMUs (Figure 1). The thigh and shank units (highlighted in the dashed box) serve as the exclusive prediction inputs. The pelvis unit acts as the trunk reference for ground-truth angle calculation; in the complete exoskeleton system, this reference sensor is placed inside the Backpack Control Module (as shown later in Figure 2) to ensure a self-contained setup. All sensors were physically sampled at 200 Hz under a unified timestamp. Each recording began with a brief upright calibration (T-pose) and included level-ground walking at two paces. All procedures followed institutional guidelines with written informed consent.

#### 3.2.2. Preprocessing Pipeline

The onboard IMU firmware applies proprietary anti-aliasing and orientation filtering, so the 200 Hz streams received by the industrial PC are already temporally smoothed at the sensor level. To align the data with the controller update rate and the prediction horizon design, all joint-angle streams were then uniformly resampled from 200 Hz to 100 Hz on the host side using linear interpolation. Angular velocity and acceleration were obtained through numerical differentiation (first- and second-order gradients) of the resampled angles. Finally, each kinematic channel was standardized using statistics computed from the training split and applied unchanged to the validation and test sets. No subject-specific tuning or additional offline filtering was introduced.

#### 3.2.3. Feature Construction and Windowing

After downsampling to 100 Hz, a rolling buffer of length *N* formed the input window $X_{t-N+1:t}\in R^{N\times 6}$ by stacking healthy-side hip and knee angle, angular velocity, and angular acceleration:

(7) $$X_{t-N+1:t}=\left[\;x_{t-N+1},\;\ldots ,\;x_{t}\;\right]\in R^{N\times 6}.$$

(8) $$\begin{array}{cc} x_{\tau } & ={\left[\theta_{H}^{\mathrm{hip}}\left(\tau \right),\;{\dot{\theta }}_{H}^{\mathrm{hip}}\left(\tau \right),\;{\ddot{\theta }}_{H}^{\mathrm{hip}}\left(\tau \right),\;\theta_{H}^{\mathrm{knee}}\left(\tau \right),\;{\dot{\theta }}_{H}^{\mathrm{knee}}\left(\tau \right),\;{\ddot{\theta }}_{H}^{\mathrm{knee}}\left(\tau \right)\right]}^{⊤},\\ \; & \;\tau \in \{t-N+1,\ldots ,t\}. \end{array}$$

The prediction target $Y_{t+1:t+M}\in R^{M\times 2}$ comprised contralateral hip and knee angles. Training samples were generated offline by sliding the window with stride s=1, whereas streaming inference updated the same buffer causally in real time. Data were partitioned by subject into training/validation/test sets (e.g., 70/15/15) to assess cross-subject generalization, and all preprocessing parameters (*N*, *M*, *s*, differentiator window/order, and normalization statistics) were fixed a priori and shared across splits.

### 3.3. Proposed Model: FusionTCN–Attention

Human gait exhibits rhythmically coupled yet asymmetric dynamics between contralateral limbs. For cooperative assistance, the predictor must be strictly causal, phase-consistent, and numerically stable at short preview horizons. The proposed FusionTCN–Attention addresses these requirements by combining a dilated causal encoder with a lightweight temporal attention fusion and joint-specific lightweight linear heads. Unlike recurrent predictors (LSTM/Seq2Seq) that accumulate phase drift via recursive states, and fully self-attentive models whose quadratic complexity undercuts deployment, this design preserves deterministic timing while adaptively focusing on phase-critical segments of motion.

Causal dilated encoder.

Let the healthy-side temporal window be $Z^{\left(0\right)}=X_{t-N+1:t}\in R^{N\times 6}$, with six kinematic channels per frame. The encoder stacks *L* residual causal blocks with exponentially increasing dilations $d_{l}=2^{\;l-1}$ and kernel size *k*. Each block expands the temporal coverage without leaking future information:

(9) $$Z^{\left(l\right)}=\mathrm{BN}\;\left(\mathrm{GELU}\;\left({\mathrm{Conv}}_{\mathrm{causal}}\left(Z^{\left(l-1\right)};k,d_{l}\right)\right)\right)+Z^{\left(l-1\right)},\;l=1,\ldots ,L.$$

During training, dropout is applied within each convolutional block to improve generalization and is disabled during inference. With this design, the encoder has an effective receptive field

(10) $$R=1+\left(k-1\right)\;\left(2^{L}-1\right).$$

With the configuration used in this study (L=5, k=3), the resulting receptive field computed from (10) is 63 frames, corresponding to approximately 0.63 s at a sampling rate of 100 Hz.

Multi-head temporal attention design and phase-capturing behavior.

In the actual implementation, the temporal attention module adopts four independent causal heads. Let $H\in R^{N\times D}$ denote the encoder output with *D* channels. For the *i*-th head, query, key, and value projections are computed as

(11) $$Q_{i}=HW_{Q,i},\;K_{i}=HW_{K,i},\;V_{i}=HW_{V,i},$$

with per-head dimensionality $d_{h}=D/4$. A causal mask $M_{\mathrm{causal}}$ is added to prevent access to future timestamps, yielding

(12) $$A_{i}=\mathrm{Softmax}\;\left(\frac{Q_{i}K_{i}^{⊤}}{\sqrt{d_{h}}}+M_{\mathrm{causal}}\right)V_{i},$$

where ${\left(M_{\mathrm{causal}}\right)}_{uv}=0$ if v≤u and −∞ otherwise. The four heads are then concatenated and linearly projected:

(13) $$A=\mathrm{Concat}\left(A_{1},\ldots ,A_{4}\right)\;W_{o}.$$

A channel-wise reweighting block (SE layer) is applied to the aggregated attention features, modulating the relative importance of each channel

(14) $$s=\sigma \;\left(W_{2}\;\mathrm{ReLU}\left(W_{1}\;\mathrm{AvgPool}\left(A\right)\right)\right),\;\tilde{A}=s⊙A,$$

where $s\in R^{D}$ encodes channel importance and ⊙ denotes elementwise multiplication.

In practice, different heads tend to emphasize distinct temporal patterns: some respond strongly near heel-strike and toe-off (large derivatives), while others capture mid-stance regularities and longer-range periodicity. This multi-head, causally masked design provides phase-aware feature aggregation under real-time constraints and supports stable contralateral trajectory prediction.

Dual-head decoding with biomechanical differentiation.

Only the most recent *M* embeddings are required for the preview:

(15) $$\tilde{H}=\mathrm{Slice}\;\left(H;\;N-M+1:N\right)\in R^{M\times D}.$$

The resulting feature matrix $\tilde{H}$ is decoded frame-wise by joint-specific lightweight linear heads,

(16) $${\hat{Y}}_{t+\tau }=\left[\begin{array}{c} W_{\mathrm{hip}}\\ W_{\mathrm{knee}} \end{array}\right]{\tilde{H}}_{\tau }+\left[\begin{array}{c} b_{\mathrm{hip}}\\ b_{\mathrm{knee}} \end{array}\right],\;\tau =1,\ldots ,M,$$

where the hip branch is deliberately smoother for torque stability, and the knee branch is assigned a higher auxiliary loss weight to accommodate larger excursions and faster dynamics—consistent with proximal–distal asymmetry.

Control-oriented composite objective.

To ensure kinematic fidelity and prevent hazard-inducing artifacts (e.g., toe-stubbing risks), we employ a composite loss function that explicitly balances trajectory tracking, smoothness, and morphological constraints:

(17) $$L_{\mathrm{total}}=\lambda_{\mathrm{mse}}L_{\mathrm{MSE}}+\lambda_{\mathrm{vel}}L_{\mathrm{vel}}+\lambda_{\mathrm{peak}}L_{\mathrm{peak}}+\lambda_{\mathrm{aux}}L_{\mathrm{aux}}.$$

where, $L_{\mathrm{MSE}}$ is the standard mean squared error governing the global trajectory. To suppress high-frequency jitter which can destabilize exoskeleton controllers, we impose a first-order velocity consistency penalty:

(18) $$\nabla_{t}{\hat{Y}}_{t}={\hat{Y}}_{t}-{\hat{Y}}_{t-1},\;L_{\mathrm{vel}}=\frac{1}{M}\sum_{t=1}^{M}{∥\nabla_{t}{\hat{Y}}_{t}-\nabla_{t}Y_{t}∥}_{2}^{2}.$$

Crucially, standard MSE often tends to “undershoot” extremum points (peaks and valleys) due to averaging effects. For gait rehabilitation, accurate peak knee flexion is vital for toe clearance. We therefore introduce a specific Peak Loss term:

(19) $$L_{\mathrm{peak}}=\frac{1}{\left|P\right|}\sum_{t\in P}{∥{\hat{Y}}_{t}-Y_{t}∥}_{2}^{2},$$

where *P* denotes the set of indices corresponding to the local extrema (top-*k* amplitude frames) within the prediction horizon. Here, $L_{\mathrm{aux}}$ increases the relative contribution of the knee joint error. The weighting coefficients (λ) were determined empirically through sensitivity analysis (see Table 1) to establish an optimal trade-off between tracking precision and morphological preservation.

### 3.4. Training and Implementation Details

To ensure reproducibility and facilitate fair comparison, all models were trained and evaluated under a unified configuration. Table 1 summarizes the key hyperparameters and implementation settings used throughout this study, including temporal windowing, network depth, optimization strategy, and hardware environment.

**Table 1:** Reproducibility configuration of the FusionTCN–Attention implementation.

Configuration Item	Value
Sampling rate	$f_{s}=100$ Hz (downsampled from 200 Hz)
Input/horizon/stride	N=250, M=40, s=1
Encoder (USE)	L=5, k=3, dilation [1,2,4,8,16], width 96
Attention fusion	H=4, embed dim D=128 (head dim 32), multi-head attention + SE gating
Loss weights	$\lambda_{mse}=1.0$, $\lambda_{peak}=0.05$, $\lambda_{vel}=0.10$, $\lambda_{aux}=0.05$
Optimizer	AdamW (weight decay $10^{-4}$, $\mathrm{lr}=10^{-3}$)
Schedule/stopping	fixed learning rate, early stopping (patience 40 epochs)
Batch size & epochs	batch 192, maximum 300 epochs
Training Hardware/software	PyTorch 2.5, CUDA 12.1, NVIDIA RTX 4070 Ti (Training Only)

These settings were fixed across all experiments to ensure reproducibility during offline training and evaluation. The hardware configuration listed above does not represent the real-time deployment platform of the exoskeleton system.

### 3.5. System Integration and Closed-Loop Real-Time Validation

Real-time validation of the proposed FusionTCN–Attention model was conducted on a unilateral lower-limb exoskeleton platform to assess system integration feasibility, closed-loop execution characteristics, and timing compatibility under physical hardware constraints. This section focuses on engineering validation rather than clinical performance.

The experimental platform is shown in Figure 2. The backpack control module (visible in Figure 2b) houses the central IMU used as the trunk reference, ensuring a self-contained sensing setup consistent with the protocol in Section 3.2.1. A unilateral lower-limb exoskeleton with two actuated joints (hip and knee) was used as a representative cooperative assistance system. Each joint was equipped with incremental encoders for position feedback and driven by embedded motor controllers. A CAN-based bus connected the joint controllers to a host computer for sensor acquisition, model inference, and reference generation. Real-time deployment was conducted on a CPU-only platform (AMD Ryzen 7 6800H, 16 GB RAM) without discrete GPU acceleration, with all inference executed online.This setup reflects a practical deployment scenario for assistive and rehabilitation systems, where compact industrial PCs or laptop-class processors are commonly used.

During cooperative walking trials, IMU data from the healthy limb were streamed to the host computer at 200 Hz and uniformly downsampled to 100 Hz to match the controller update rate. A rolling temporal buffer of length N=250 frames was continuously maintained. At each control cycle, the buffer was processed by the FusionTCN–Attention model to generate a short-horizon contralateral joint trajectory prediction with a horizon of M=40 frames. The same temporal configuration and preprocessing pipeline were used during offline training and online inference, ensuring consistency between learning and deployment.

**Figure 3:**
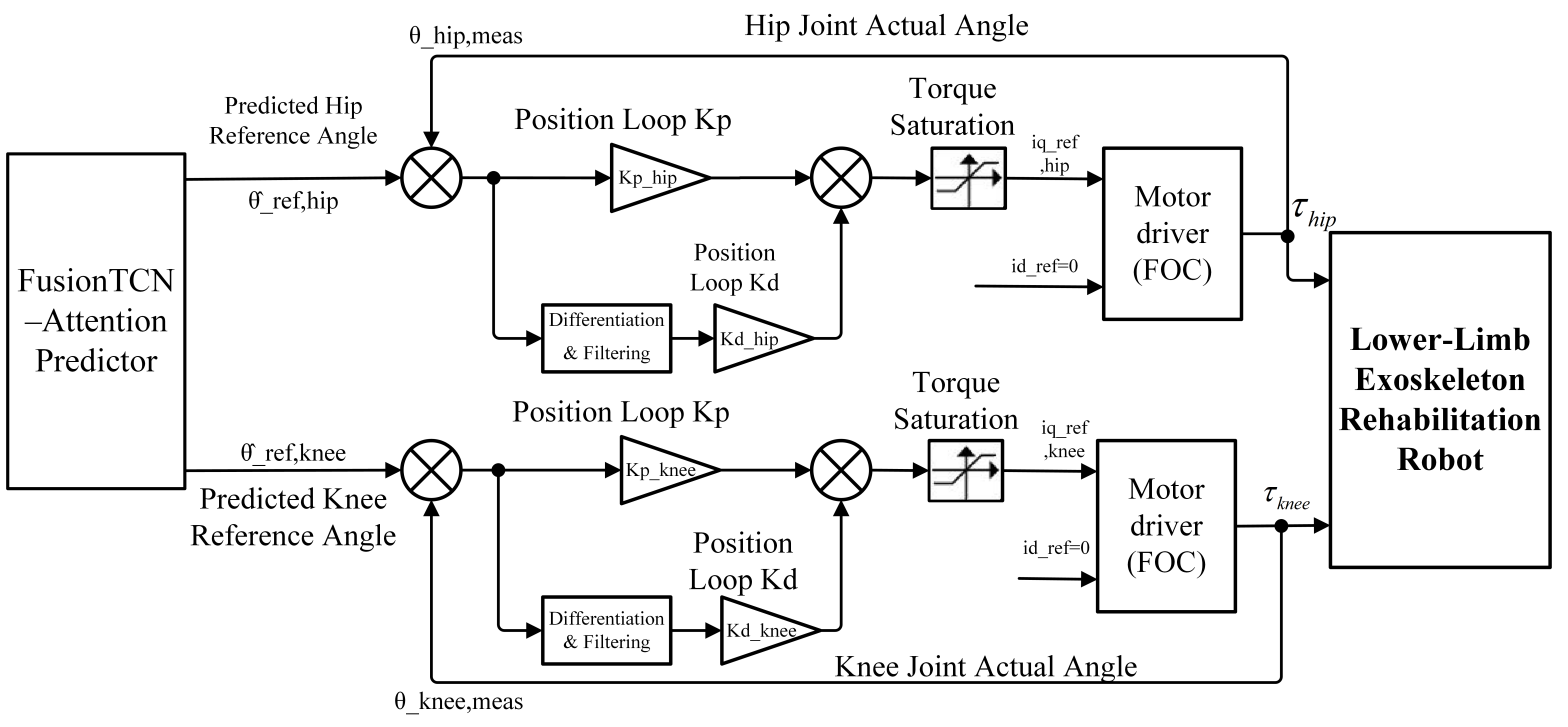
Closed-loop control architecture of the unilateral exoskeleton.

The control architecture adopted for real-time validation is illustrated in Figure 3. The predicted joint trajectories were used exclusively as reference inputs within a feedback-controlled system. At each control cycle, only the next-step prediction ${\hat{Y}}_{t+1}$ was supplied to the low-level joint controller. Joint actuation was governed by a PD controller with encoder feedback, while subsequent predicted frames were used to shape the feedforward reference profile. As a result, joint motion was continuously regulated by feedback, and prediction errors did not accumulate across control cycles.

Standard hardware safety constraints were enforced throughout all real-time experiments. These included joint-level torque and velocity saturation at the motor-controller level, watchdog-based controller monitoring, and a hardware emergency-stop circuit. All experiments were conducted on healthy adult subjects under human supervision. Extension of the proposed framework to impaired users would require additional safety certification and clinical validation, which are beyond the scope of this study.

## 4. Results

### 4.1. Evaluation Protocol and Metrics

To evaluate the proposed causal prediction framework under conditions that closely resemble real deployment, unilateral gait data were collected from twenty-one adult volunteers walking along a straight indoor corridor at two self-selected paces (Normal and Fast). All inertial signals were physically sampled at 200 Hz and uniformly resampled to 100 Hz, matching the control frequency of the cooperative exoskeleton described in Section 3.2. Three IMUs were attached to the healthy-side pelvis, thigh, and shank. Pelvis measurements were used exclusively to reconstruct reference joint angles, whereas the prediction architectures received only thigh and shank streams, mirroring the real-time configuration of the assistive system.

A subject-exclusive split was adopted (15/3/3 for training/validation/testing) to prevent cross-subject leakage. Unless otherwise stated, the temporal context window was fixed at N=250 frames (2.5 s) and the prediction horizon at M=40 frames (0.4 s), with stride s=1 for frame-wise evaluation. Performance was computed separately for hip and knee and summarized across paces.

Model assessment combined pointwise accuracy with rhythm-preserving fidelity. Trajectory agreement was quantified by root-mean-squared error (RMSE) and Pearson correlation coefficient

(20) $$\mathrm{RMSE}=\sqrt{\frac{1}{M}\sum_{i=1}^{M}{\left({\hat{y}}_{i}-y_{i}\right)}^{2}},\;r=\mathrm{corr}\left(\hat{y},y\right).$$

Temporal consistency was assessed by a velocity-domain RMSE (vRMSE) computed on first-order differences Δy. Stride-scale morphology was summarized by the peak amplitude error (PeakAmpErr, degrees). Phase alignment was characterized by PhaseLag (%), defined as the time shift that maximizes the normalized cross-correlation and normalized by the median gait period. Model size is reported as trainable parameters (millions).

### 4.2. Quantitative Benchmark Comparison

Four causal predictors—FusionTCN–Attention, Seq2Seq, PlainTCN, and a masked self-attention Transformer—were evaluated under identical splits, windowing, and optimization. Table 2 lists angle errors (°), correlations, absolute lag (ms), peak-amplitude error (°), and parameter counts (M).

Overall, FusionTCN–Attention attains the lowest angle errors and highest correlations with a compact footprint (hip RMSE 5.71°, knee RMSE 7.43°, $r_{Hip}$ 0.905, $r_{Knee}$ 0.912, AbsLag 14.56 ms, PeakErr 1.99°, Params 0.366 M). The Transformer narrows the gap in correlation but exhibits larger peak deviations (PeakErr 4.27°). PlainTCN remains competitive on knee RMSE (7.29°) at modest lag (12.45 ms) but shows residual over-/undershoot near extrema. Seq2Seq presents the largest RMSEs (hip 9.50°, knee 10.06°) and substantially higher lag (32.26 ms), accompanied by significant peak attenuation (PeakErr 6.92°), which is consistent with the multi-frame phase drift observed at faster cadences.

**Figure 4:**
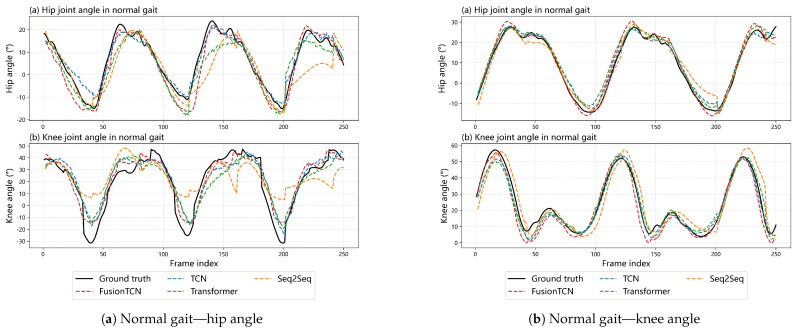

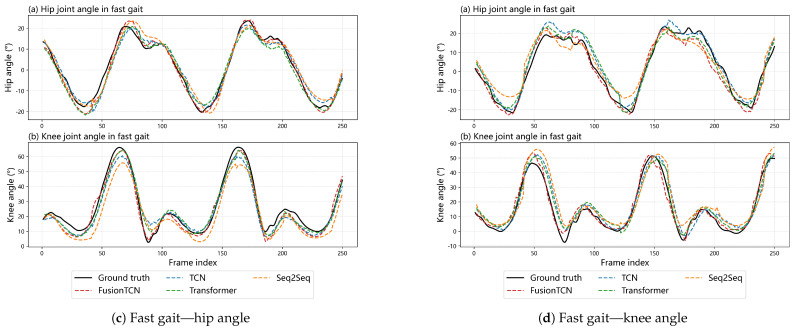
Comparison of predicted joint angles under Normal and Fast walking. Normal gait: hip (**a**), knee (**b**); Fast gait: hip (**c**), knee (**d**).

Figure 4 shows long-sequence hip and knee angle predictions under Normal and Fast walking. FusionTCN—Attention exhibits the closest temporal alignment with the ground truth across cycles, with minimal phase drift and limited peak attenuation, especially around heel—strike and toe—off. Seq2Seq shows clear phase lag and peak attenuation over longer horizons, which is particularly detrimental for real-time gait assistance, as accumulated temporal misalignment degrades cycle-level synchronization, while PlainTCN and the Transformer present moderate overshoot or amplitude bias near extrema, consistent with the quantitative trends in Table 2.

### 4.3. Ablation and Design Sensitivity

A controlled ablation on FusionTCN–Attention modified exactly one factor per variant while holding all else fixed. The A-series removed structural components (attention, fusion head, residual behavior, or causality); the B-series adjusted loss components. Table 3 and Table 4 report means across test subjects.

Removing temporal attention (A1) increases both angle errors and lag, indicating a role in timing stability during rapid transitions. Disabling fusion (knee-only head, A2) compromises cross-joint consistency by construction and lowers knee-side correlation relative to the full model. Approximating residual removal by feature de-meaning (A3) degrades fit, suggesting residual flows help preserve high-slope information. A non-causal TCN (A4) achieves strong offline scores (e.g., hip RMSE 5.28°) yet violates deployable causality, hence it is excluded from real-time candidates.

Loss-oriented variants reveal complementary roles of objective terms (Table 4). A pure MSE (B1) raises errors and lag, showing that dynamics-aware components add value beyond pointwise fit. Disabling the auxiliary knee term (B2) prioritizes knee−side fidelity (best knee RMSE and $r_{\mathrm{Knee}}$) and lowers peak error, indicating a tunable trade-off for joint-specific accuracy. Removing the velocity term (B) yields the best hip−side fit and the smallest lag, reflecting a shift from trajectory smoothness to pointwise agreement. Eliminating the peak term (B4) markedly inflates PeakErr, confirming its necessity for stride-scale morphology.

### 4.4. Temporal Error Profiles and Phase Consistency

**Figure 5:**
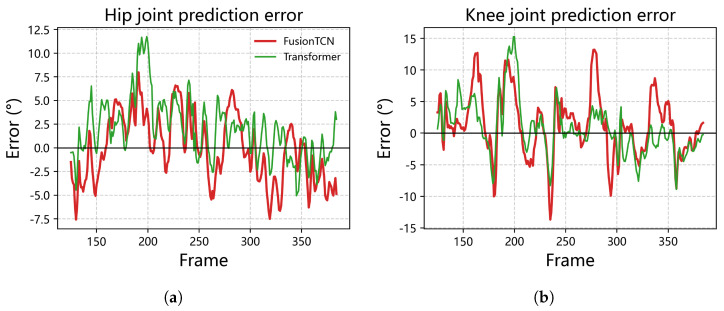
Long−sequence residuals for hip and knee joints (degrees). (**a**) Hip joint prediction error over time. (**b**) Knee joint prediction error over time. The horizontal line indicates zero error.

Beyond aggregates, error time-courses and state-space geometry were examined to verify adherence to gait dynamics. Figure 5 displays frame-wise residuals (prediction−ground truth, degrees) over long sequences. FusionTCN–Attention forms a narrow, near-zero envelope: the mean trace stays within ±3° for most segments, with brief troughs around −5° near rapid reversals. The plain causal TCN shows wider excursions (typically ±5°). Seq2Seq exhibits intermittent bursts exceeding 8° around fast flexion/extension, aligning with its larger RMSEs and higher lag; the transformer narrows the correlation gap yet still shows 4–6° drift at slope extremes, consistent with its higher PeakErr.

**Figure 6:**
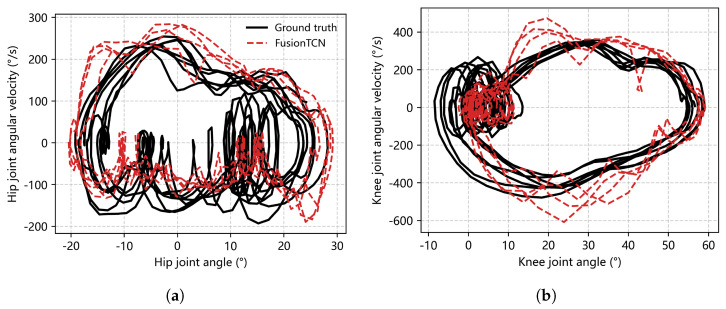
Hip and knee joint phase portraits. (**a**) Hip joint phase portrait: angular velocity versus joint angle. (**b**) Knee joint phase portrait: angular velocity versus joint angle. Ground truth is shown in black, and FusionTCN–Attention predictions are shown in red.

Angle−velocity phase portraits (Figure 6) probe limit-cycle fidelity. Ground-truth hip loops span roughly [−20°,30°] and [−200,300]°/s; knee loops cover about [−10°,60°] and [−600,450]°/s. FusionTCN−Attention preserves loop shape and orientation with minimal distortion: peak angular-velocity loci align within one grid step (≈50–100°/s) without spurious self-intersections; models lacking temporal attention or using recurrent decoding display thinning/ballooning near apices, consistent with larger PeakErr and drift.

**Figure 7:**
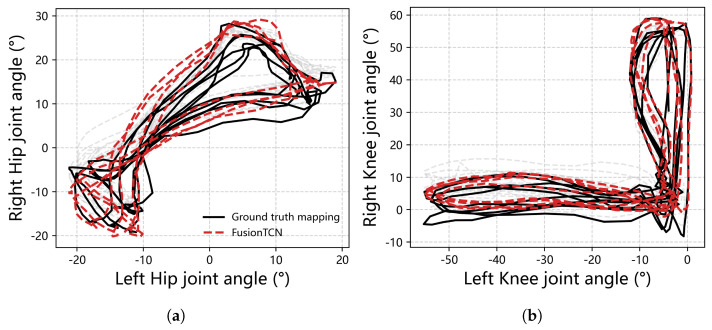
Unilateral angle−angle loops during gait: (**a**) hip joint loop (right hip versus left hip angle); (**b**) knee joint loop (right knee versus left knee angle). Ground truth trajectories are shown in black, and FusionTCN predictions in red.

Bilateral angle−angle loops (Figure 7) assess inter-limb coupling. For the hip, the traversed corridor is about 20° wide; FusionTCN–Attention stays inside this corridor with cycle-wise bias of only a few degrees. For the knee, the narrow loop around mid-stance is particularly sensitive; predictions adhere without systematic hysteresis widening, consistent with the small PeakErr in Table 2.

**Figure 8:**
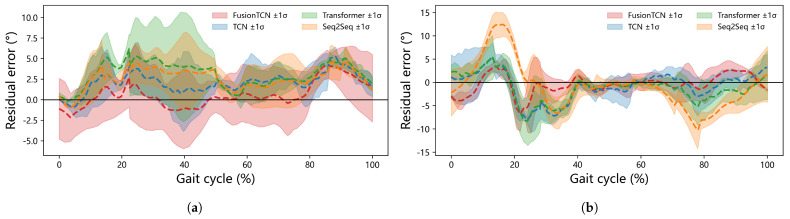
Residual error distributions across normalized gait cycles for (**a**) hip and (**b**) knee joints among different models. Shaded regions denote mean ±σ of residuals across test samples at each normalized gait phase.

Cycle-normalized residual ribbons (Figure 8) summarize mean error and ±1σ bands over 0–100% gait. For the hip (a), FusionTCN−Attention maintains a near-zero mean from ∼55–85% with bands largely within ±3°; the largest negative swing (≈−5°) appears around 35–45% near push-off. For the knee (b), the mean residual remains close to zero over mid-stance and late swing, with a brief negative dip of ∼6–8° near ∼25–30% where slope changes are steep. In contrast, Seq2Seq shows a pronounced trough near 75–80% (exceeding 10°), matching its higher RMSE and lag. These phase-wise trends align with Table 2.

### 4.5. Real-Time Validation

**Figure 9:**
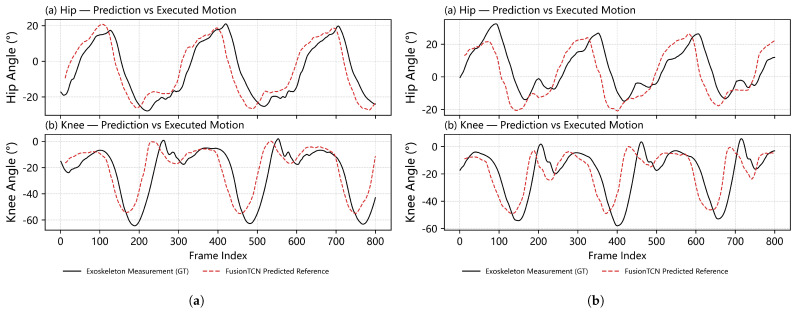
Real−time tracking of hip and knee joint angles during unilateral exoskeleton assistance. Solid black curves denote exoskeleton encoder measurements, and red dashed curves denote FusionTCN–Attention predicted references; (**a**) walking trial 1 (**b**) walking trial 2.

Finally, FusionTCN–Attention was deployed on the unilateral exoskeleton platform for hardware-in-the-loop real-time validation. During closed-loop operation at 100 Hz, the end-to-end delay from IMU sampling to actuator command generation, including sensing, communication, inference, and motor control, remained within the 10 ms control cycle budget. No instability, sustained oscillation, or perceptible delay was observed during repeated walking trials. Representative long-sequence overlays recorded under wear conditions (Figure 9) show that the predicted reference trajectories closely follow the executed hip and knee motions over consecutive gait cycles, with accurate timing around heel-strike and toe-off.

## 5. Discussion

The proposed FusionTCN–Attention demonstrates accurate contralateral prediction suitable for real-time assistance. Table 2 reports hip and knee RMSEs of 5.71° and 7.43° with high correlations (r>0.9). The mean absolute lag of 14.56 ms falls within a single 100 Hz control cycle, minimizing controller phase compensation requirements. Contrastingly, the Transformer model exhibits higher peak errors (4.27°), and PlainTCN shows oscillation near signal peaks. The Seq2Seq baseline yields the highest errors and a 32.26 ms lag, confirming phase drift accumulation in recursive decoding. Long-sequence plots (Figure 4) verify the model’s capability to track rapid trajectory changes during toe-off and heel-strike.

Component−wise ablation validates the architectural design choices. Removing temporal attention (A1) degrades all accuracy metrics and increases latency, confirming its role in stabilizing timing during transitions. The knee-only fusion variant (A2) results in the highest system latency (22.67 ms), demonstrating that cross-joint feature integration is critical for minimizing phase lag. Approximating residual removal (A3) yields higher angle errors, suggesting that residual flows are essential for preserving high-slope information. While the non-causal TCN (A4) achieves lower hip error, it degrades knee tracking accuracy compared to the proposed causal model and violates real-time constraints. Regarding loss components, the full composite objective (A0) represents the optimal trade-off. Disabling the auxiliary knee term (B2) or the velocity term (B3) effectively reduces latency and peak error but compromises global tracking accuracy (increasing RMSE relative to the full model). Finally, excluding the peak-oriented term (B4) more than doubles the peak error (4.95° vs. 1.99°), validating its necessity for maintaining stride-scale morphology.

Limitations of this study are noted regarding the dataset. The evaluation utilizes data from unimpaired adults as an engineering benchmark to verify algorithmic causality and stability. The trunk reference frame captures mechanically valid kinematics for both symmetric and asymmetric gait. The use of healthy training data represents a deliberate design choice to generate “normative” reference trajectories that guide the paretic limb toward a corrected pattern, avoiding the replication of pathological asymmetry. Post-stroke hemiparesis involves spasticity and compensatory movements distinct from healthy coupling; consequently, prediction errors in clinical populations may exceed current reports. Future work will use this model as a pre-trained baseline for transfer learning on patient-specific datasets.

### 5.1. Clinical Translation and Hardware Integration

Clinical deployment requires seamless algorithm–hardware integration. The model’s lightweight architecture (0.366 M parameters) suits embedded platforms in wearable robotics, such as the STM32 microcontroller or NVIDIA Jetson series. Future implementations will integrate confidence-based gating to ensure safety; if predictive uncertainty exceeds a threshold (e.g., during unobserved movements), the controller decays to a transparent mode. Consistent, deterministic prediction facilitates long-term user adaptation and potentially reduces metabolic cost as the user learns to trust the assistance.

### 5.2. Biomimetic Extensions and Environmental Adaptation

Future work will focus on biomimetic extensions mirroring central nervous system adaptation to varying contexts. For variable cadence, the fixed-window approach can incorporate phase-variable estimation, aligning the TCN receptive field with gait cycle percentage rather than absolute time. For terrain adaptation (e.g., stairs, ramps), a proposed multi-modal fusion framework supplements IMU data with vision-based context or EMG signals. This mimics biological sensorimotor integration, allowing preemptive impedance adjustment for stair ascent or descent to overcome level-ground limitations.

## 6. Conclusions

This paper presents FusionTCN–Attention, a causal temporal modeling framework designed for real-time contralateral gait prediction. By integrating a dilated causal Convolutional Network with a temporal attention mechanism, the architecture captures long-range gait dependencies while enforcing the strict causality required for closed-loop control. Drawing on the biomechanical principle of contralateral coupling, the proposed method reconstructs hip and knee trajectories using a minimal unilateral IMU setup.

Experimental validation on healthy subjects demonstrates the model’s tracking accuracy and timing stability. The method achieves hip and knee RMSEs of 5.71° and 7.43° (r>0.9), showing improved waveform fidelity and peak preservation compared to Seq2Seq and Transformer baselines. Furthermore, the model maintains a deterministic phase lag of 14.56 ms within a 100 Hz control cycle, effectively mitigating the phase drift issues observed in recurrent architectures.

From an engineering perspective, the study verifies the feasibility of high-fidelity gait estimation under embedded hardware constraints. With 0.366 M parameters, the model offers a lightweight module suitable for deployment on microcontroller-based exoskeleton platforms without GPU acceleration. Future work will focus on extending this baseline to hemiparetic cohorts via transfer learning and incorporating environmental adaptation strategies.

## Figures and Tables

**Table 2:** Prediction comparison across models on a representative trial at 100 Hz.

Model	Hip RMSE (°)	Knee RMSE (°)	$r_{\mathrm{Hip}}$	$r_{\mathrm{Knee}}$	AbsLag (ms)	PeakErr (°)	Params (M)
**FusionTCN (ours)**	5.71	7.43	0.905	0.912	14.56	1.99	0.366
Seq2Seq	9.50	10.06	0.848	0.854	32.26	6.92	0.052
PlainTCN	7.38	7.29	0.890	0.915	12.45	3.88	0.622
Transformer	6.56	7.25	0.883	0.911	12.43	4.27	0.558

**Table 3:** Ablation of FusionTCN−Attention (A−series; mean across test subjects). AbsLag (ms) is the mean absolute lag across hip and knee; A2 uses knee only.

Variant	Hip RMSE (°)	Knee RMSE (°)	$r_{\mathrm{Hip}}$	$r_{\mathrm{Knee}}$	AbsLag (ms)	PeakAmpErr (°)	Params (M)
A0 Full Model	5.71	7.43	0.905	0.912	14.56	1.99	0.366
A1 No Attention	6.40	8.85	0.908	0.870	16.55	2.82	0.326
A2 No Fusion (knee-only head)	14.92	7.81	—	0.896	22.67	3.08	0.366
A3 No Residual (approx.)	6.89	8.20	0.904	0.889	17.58	4.53	0.366
A4 Non-causal TCN	5.29	7.94	0.917	0.900	14.88	1.37	0.366

**Table 4:** Ablation of FusionTCN–Attention (B-series: loss-component removals; mean across test subjects). AbsLag (ms) is the mean absolute lag across hip and knee.

Variant	Hip RMSE (°)	Knee RMSE (°)	$r_{\mathrm{Hip}}$	$r_{\mathrm{Knee}}$	AbsLag (ms)	PeakAmpErr (°)	Params (M)
B1 MSE-only	6.17	8.09	0.911	0.891	14.65	2.65	0.366
B2 No KneeAux	6.37	7.47	0.911	0.912	11.48	0.92	0.366
B3 No Velocity	5.78	7.93	0.915	0.899	11.74	1.67	0.366
B4 No Peak	6.51	7.82	0.895	0.897	16.72	4.95	0.366

## Data Availability

Data is unavailable due to privacy or ethical restrictions.

## Abbreviations

The following abbreviations are used in this manuscript:

IMU	Inertial Measurement Unit
TCN	Temporal Convolutional Network
RMSE	Root Mean Square Error
Seq2Seq	Sequence-to-Sequence
LSTM	Long Short-Term Memory
HS	Heel-Strike
TO	Toe-Off
SE	Squeeze-and-Excitation
SEMG	Surface Electromyography
FOC	Field-Oriented Control
